# Incidence and mortality of cervical cancer in French Guiana: Temporal and spatial trends

**DOI:** 10.1016/j.puhip.2021.100138

**Published:** 2021-05-16

**Authors:** Laure Manuella Imounga, Juliette Plenet, Sophie Belliardo, Elie Chow Chine, Antoine Adenis, Mélanie Gaillet, Nadia Thomas, Céline Michaud, Véronique Servas, Pierre Couppié, Kinan Drak Alsibai, Mathieu Nacher

**Affiliations:** a Registre des Cancers de la Guyane, URPS, 97300, Cayenne, French Guiana; b CIC INSERM 1424, Centre hospitalier Andrée Rosemon Cayenne, 97300, Cayenne, French Guiana; c DFR Santé, Université de Guyane, Cayenne, 97300, Cayenne, French Guiana; d Centres délocalisés de prévention et de soins, Centre hospitalier de Cayenne, 97300, Cayenne, French Guiana; e Department of dermatology, Centre hospitalier Andree Rosemon Cayenne, 97300, Cayenne, French Guiana; f Department of Pathology, Centre Hospitalier Andrée Rosemon, 97300, Cayenne, French Guiana

**Keywords:** Cervical cancer, Incidence, Mortality, Latin America, HPV

## Abstract

**Objectives:**

Cervical cancer is the second most frequent cancer among women in French Guiana. The objective was to review a decade of cervical cancer data, and to study spatial and temporal trends.

**Study design:**

The design was retrospective and descriptive.

**Methods:**

The cancer registry of French Guiana compiled exhaustive data on cervical cancer throughout French Guiana between 2005 and 2015. Age-standardized incidence and mortality were computed and mapped to identify priority areas.

**Results:**

With 232 new cases recorded in French Guiana between 2005 and 2014 (23 annual cases), cervical cancer ranked 5th among all incident cancers (11%) and was the 2nd most frequent cancer in women (12% of cancers among women). The standardized incidence rate over the period 2005–2014 was 23.8 cases of cervical cancer per 100 000 woman-years. Between 2005-2009 and 2010–2014 the incidence of cervical cancer decreased from 26.26 cases per 100 000 to 22.66 cases per 100 000 and the mortality rate from cervical cancer decreased from 6 deaths per 100 000 to 3.2 deaths per 100 000.

Within French Guiana, the standardized incidence rates were very heterogenous with the highest rates in remote areas. The standardized death rate from cervical cancer over the 2005–2014 decade was 4.4 cases per 100 000 woman-years.

**Conclusions:**

The present results suggest there has been progress in French Guiana, but there are still areas where screening is challenging and should be expanded. The recent authorization of HPV testing is an opportunity that could help health professionals achieve this goal. HPV vaccination –with a nonavalent vaccine—is also an important public health endeavor that could alleviate the burden of cervical cancer among the cohorts of women benefitting from it.

## Highlights

• In French Guiana, cervical cancer is the second most frequent cancer among women.
• The incidence of cervical cancer was the second highest in Latin America but mortality was the lowest.
• The incidence and mortality of cervical cancer has decreased in the past decade.
• Cervical cancer incidence was spatially heterogeneous: higher in remote areas where HPV prevalence is high and specialized care scarce.
• Operational priorities for prevention and screening should have a special focus on remote areas.

## 1 Introduction

Despite the shift from the burden from infectious diseases towards chronic non communicable diseases, infections still remain a major cause of morbidity and mortality in the tropics. Infectious agents causing chronic infections are also important causes of cancer. In sub-Saharan Africa, it is estimated that 1/3 of all cancers are caused by infections, and that in developing countries, controlling these infections could avoid up to a quarter of all cancers [[1], [2], [3], [4]]. Human papillomaviruses (HPV) cause 5.2% of the global burden of cancer and in developing countries the proportion increases to 7.7%. Cervical cancer represents the second most frequent cancer in women in French Guiana. It has also been an important cause of mortality[5,6]. Most of the French Guianese territory is covered by primary forest, hence population density is low. For the 20% of the population that are living in remote parts of the territory, there is a network of health centers that are coordinated by the main hospital. However, accessing care can be difficult for those who do not live in the main villages. Because of the early sex initiation, and perhaps cultural practices of vaginal steam baths and dry sex, women living in remote areas are at greater risk of cervical lesions due to HPV[7]. While information on what HPV genotypes are present in cancer samples is presently unknown in French Guiana, we have shown that among women living on the Maroni and Oyapock rivers, the prevalence of HPV was very high, and that HPV testing found different genotypes from what is observed in mainland France, an observation that justified the use of nonavalent vaccines to prevent cervical cancer in these remote areas of high prevalence[[8], [9], [10]]. Moreover, the proportion of abnormal smears was higher than what is observed in mainland France. We observed that 10% of women had cytological anomalies, double of what has been reported in Amazonas state in Brazil[11]. Among cervicovaginal smear, 1.2% had HSIL, a figure four times what was observed in Paris in 2002[12]. After the first reports from the cancer registry of French Guiana on 2003–2005, cervical cancer has been the focus of increased screening efforts, notably in remote areas and vulnerable populations. Here our objective was to review a decade of cervical cancer data, and to study spatial and temporal trends.

## 2 Methods

The Registre du Cancer de Guyane (RCG) conducts a regular inventory –aiming at exhaustivity– of the epidemiology of cancer in French Guiana. Created in 2005, it uses hundreds of notification and information sources, mostly from outside French Guiana. This is of paramount importance because patients are often referred to large cancer centers in mainland France. In French Guiana, data collection mostly focuses on the hospitals in Cayenne, Kourou, and Saint Laurent du Maroni. The coding of tumors by research technicians follow the rules of French and European registries, FRANCIM and ENCR. New cases of cancer diagnosed from January 1, 2003, corresponding to invasive and/or *in situ* tumors of patients residing in French Guiana, are continuously registered regardless of the cancer location and location.

The Registry relies on the National Information Systems Medicalization Program data (PMSI), transmitted by FRANCIM and ATIH, which compile all hospital stays with ICD10 codes and a corresponding location code in French Guiana; this allows the query of health structures within and outside of French Guiana. Data collection also relies on Medical Information Departments (DIM) and hospital archives, and local physicians, pathologists, biologists, and NGOs involved in cancer screening.

Every year, the data is transferred to the Hospices Civils de Lyon (HCL) and to the International Agency for Research on Cancer (IARC).

For cervical cancer the ICD codes used were all the C53 codes. STATA software (STATA Corporation, College Station, Texas) was used for statistical analysis and mapping. Standardization on age structure was performed using the world population.

The registry has been approved by the National regulatory authorities (Commission Nationale Informatique et Libertés).

## 3 Results

With 232 new cases recorded in French Guiana between 2005 and 2014 (23 annual cases), cervical cancer ranked 5th among all incident cancers (11%) and was the 2nd most frequent cancer in women (12% of cancers among women) after breast cancer, ahead of colon-rectal, uterine corpus and thyroid cancers.

### 3.1 Incidence for the 2005–2014 decade

**Fig. 1:**
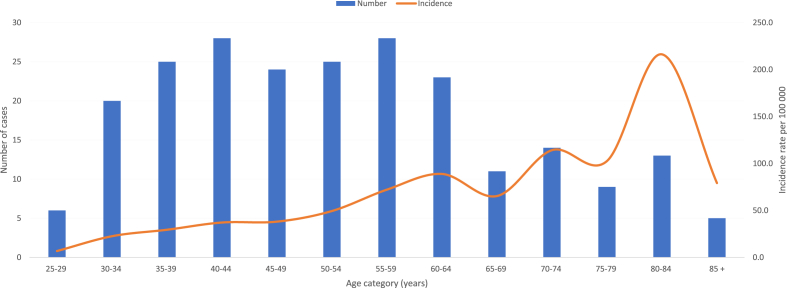
Number of cases of cervical cancer and incidence rate per age: french guiana 2005–2014.

Cervical occurred in about 78% of cases in women aged 40 (Fig. 1) and over and the median age at diagnosis was 52 years (51 years in France).

Between 2005 and 2014, the specific incidence increased gradually with age, with some fluctuations until it peaked at 80–84 years (216.2) (Fig. 1).

The standardized incidence rate over the period 2005–2014 was 23.8 cases of cervical cancer per 100 000 woman-years, whereas it is only 6.7 in France in 2012.

**Fig. 2:**
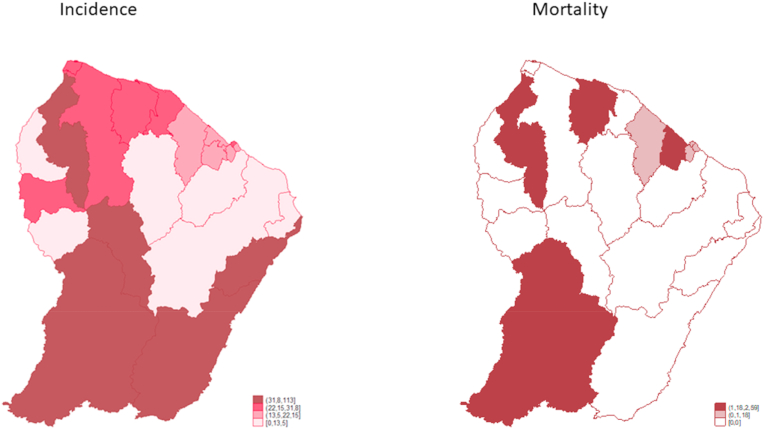
Spatial distribution of standardized incidence and mortaliy rates from cervical cancer, french guiana 2005–2014.

At the regional level, the standardized incidence rates were very heterogenous (Fig. 2) varying between 0.0 for the communes of Ouanary, Régina, Roura, Saint-Elie and 113.0 per 100 000 woman-years for Camopi (East). Nine other municipalities –also all rural– had an incidence rate above the regional average: Saül, Maripasoula, Saint-Laurent-du-Maroni, Awala-Yalimapo, Mana et Grand-Santi (West): respectively 100.0; 93.9; 35.2; 31.8; 26 and 24.9: Saint-Georges (East): 42.2; Cayenne (Centre coastal): 26.9; Iracoubo (Savanas): 26.5.

### 3.2 Mortality for the 2005–2014 decade

With 40 deaths recorded in French Guiana between 2005 and 2014 (4 annual deaths), cervical cancer ranked 8th among all cancers of all sexes combined (3.1%) and, after breast cancer, represented the 2nd deadliest cancer in women (7.2% of cancer deaths in women).

**Fig. 3:**
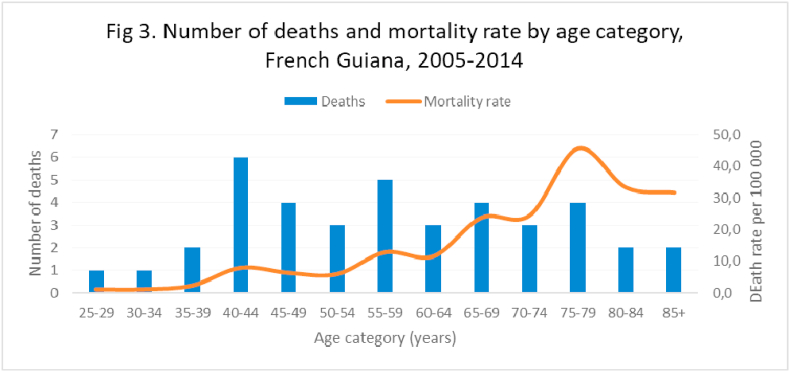
Number of deaths and mortality rate by age category, french guiana,2005–2014.

Between 2005 and 2014, the specific mortality associated with cervical cancer was low before age 40 (less than 8 cases per 100 000 woman-years) then gradually increased reaching its maximum at 75–79 years (Fig. 3). The median age at death was 58 years.

The standardized death rate from cervical cancer over the 2005–2014 decade was 4.4 cases per 100 000 woman-years, whereas, in 2012, it was 1.8 in France.

During this decade, at the regional level, the standardized mortality ratios (SMR) to the total Guyanese female population was spatially heterogenous (Fig. 2).

### 3.3 Evolution of incidence and mortality between 2005 and 2014

Between 2005-2009 and 2010–2014 the incidence of cervical cancer decreased from 26.26 cases per 100 000 to 22.66 cases per 100 000. Regarding deaths, between 2005-2009 and 2010–2014 the mortality rate from cervical cancer decreased from 6 deaths per 100 000 to 3.2 deaths per 100 000.

### 3.4 Comparison of incidence and mortality with Latin America

**Fig. 4:**
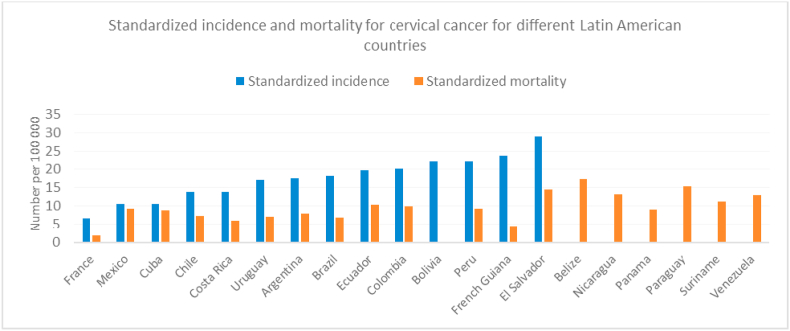
Incidence and mortality of cervical cancer in Latin America.

**Fig. 5:**
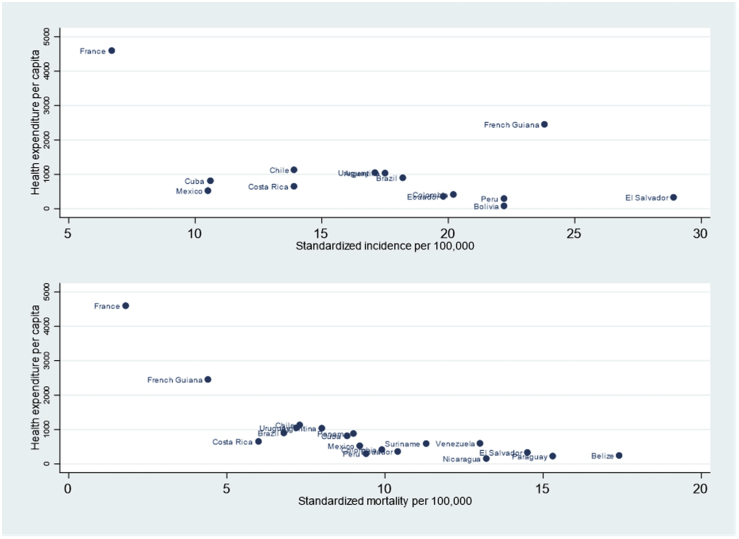
Standardized incidence and mortality of cervical cancer by Health expenditure per capita.

Fig. 4 shows the standardized incidence and mortality rates for Latin American countries, French Guiana and France. It shows French Guiana as an outlier with a high incidence rate but relatively low mortality rate. Fig. 5 shows the relation between health expenditure per capita (2014) and standardized incidence and mortality from cervical cancer. There was a non-significant negative relation between incidence and health expenditure per capita (Spearman's Rho = −0.49, P = 0.07), and a highly significant negative correlation between health expenditure per capita and mortality from cervical cancer (Spearman's Rho = −0.88, P < 0.0001; 77% of the variance explained by health expenditure per capita).

## 4 Discussion

The present results show that the incidence of cervical cancer and mortality rate from cervical declined in French Guiana. The incidence of cervical cancer is still very high for a territory with high health expenditure per capita when compared to other Latin American countries. However, although it is higher than in mainland France it is much lower than in most of Latin America. This suggests that, despite some progress, there is still insufficient screening for cytological anomalies and/or the presence of high-risk HPV genotypes. HPV testing has shown its benefits in terms of incidence and mortality from cervical cancer but in France it has only been recommended and reimbursed in 2020 [13,14]. HPV testing –notably self-sampling—will presumably allow flexibility in remote areas where health professionals are scarce and HPV prevalence is very high [8,10,15]. The spatial distribution of cervical cancer incidence and mortality in French Guiana shows that the areas where the problem is most acute are the remote areas, which poses a challenge to the health system but also suggest specific prioritization efforts if the burden of cervical cancer is to be further reduced. Furthermore, these areas are inhabited by native populations –maroons and Amerindians— who are infected by different viruses and may have different immunogenetic makeups and predispositions for cancer [16]. Regarding mortality, when compared to France mortality from cervical cancer is higher in French Guiana but when compared to other Latin American countries it is much lower, and strongly negatively correlated to per capita health expenditures.

The decline in incidence and mortality may in part result from recent efforts to improve gynecological care in French Guiana. In coastal areas, gynecologists and midwives have increased the possibility of smears; training efforts have resulted in routine smears in pregnant women; in 2012 organized screening was implemented in French Guiana. In the interior regions, the screening programme only started in 2013 (recruitment of midwives, mobile colposcopy) therefore it is too early to evaluate its impact. Women aged over 45 being less likely to consult for pregnancy or contraception are often challenging because the are more likely to have late diagnoses.

The limitations of the present study are that the small population size of French Guiana lead to small patient numbers, notably in terms of mortality. A specific problem is that of population migration, notably along the border rivers, with some cases of cancer of patients living in neighboring Suriname or Brazil are actually diagnosed in French Guiana but not counted in the registry data. Cooperation efforts and data sharing may help get a clearer view of this problem linked to the specific migratory context of French Guiana. Nevertheless, a decade of exhaustive data collected still provides valuable information to guide prevention and care efforts.

In conclusion, the present results summarizing a decade of cervical cancer data suggest there has been progress in French Guiana, but there are still areas where screening is challenging and should be expanded. The recent authorization of HPV testing is an opportunity that could help health professionals achieve this goal. HPV vaccination –with a nonavalent vaccine—is also an important public health endeavor that could alleviate the burden of cervical cancer among the cohorts of women benefitting from it.

## Author statements

Ethical and regulatory aspects. The Registre des Cancers de la Guyane is a certified registry that has been approved by the National regulatory authority the Commission Nationale Informatique et Libertés.

## Funding

None declared.

## Competing interest

The authors declare no competing interests.
